# *In vitro *comparison of antiviral drugs against feline herpesvirus 1

**DOI:** 10.1186/1746-6148-2-13

**Published:** 2006-04-26

**Authors:** K van der Meulen, B Garré, S Croubels, H Nauwynck

**Affiliations:** 1 Laboratory of Virology; 2 Department of Pharmacology, Pharmacy and Toxicology, Faculty of Veterinary Medicine, Salisburylaan 133, Ghent University, 9820 Merelbeke, Belgium

## Abstract

**Background:**

Feline herpesvirus 1 (FHV-1) is a common cause of respiratory and ocular disease in cats. Especially in young kittens that have not yet reached the age of vaccination, but already lost maternal immunity, severe disease may occur. Therefore, there is a need for an effective antiviral treatment. In the present study, the efficacy of six antiviral drugs, i.e. acyclovir, ganciclovir, cidofovir, foscarnet, adefovir and 9-(2-phosphonylmethoxyethyl)-2, 6-diaminopurine (PMEDAP), against FHV-1 was compared in Crandell-Rees feline kidney (CRFK) cells using reduction in plaque number and plaque size as parameters.

**Results:**

The capacity to reduce the number of plaques was most pronounced for ganciclovir, PMEDAP and cidofovir. IC_50 (NUMBER) _values were 3.2 μg/ml (12.5 μM), 4.8 μg/ml (14.3 μM) and 6 μg/ml (21.5 μM), respectively. Adefovir and foscarnet were intermediately efficient with an IC_50 (NUMBER) _of 20 μg/ml (73.2 μM) and 27 μg/ml (140.6 μM), respectively. Acyclovir was least efficient (IC_50 (NUMBER) _of 56 μg/ml or 248.7 μM). All antiviral drugs were able to significantly reduce plaque size when compared with the untreated control. As observed for the reduction in plaque number, ganciclovir, PMEDAP and cidofovir were most potent in reducing plaque size. IC_50 (SIZE) _values were 0.4 μg/ml (1.7 μM), 0.9 μg/ml (2.7 μM) and 0.2 μg/ml (0.7 μM), respectively. Adefovir and foscarnet were intermediately potent, with an IC_50 (SIZE) _of 4 μg/ml (14.6 μM) and 7 μg/ml (36.4 μM), respectively. Acyclovir was least potent (IC_50 (SIZE) _of 15 μg/ml or 66.6 μM). The results demonstrate that the IC_50 (SIZE) _values were notably lower than the IC_50 (NUMBER) _values. The most remarkable effect was observed for cidofovir and ganciclovir. None of the products were toxic for CRFK cells at antiviral concentrations.

**Conclusion:**

In conclusion, measuring reduction in plaque number and plaque size are two valuable and complementary means of assessing the efficacy of an antiviral drug. By using these parameters for six selected antiviral drugs, we found that ganciclovir, PMEDAP, and cidofovir are the most potent inhibitors of FHV-1 replication in CRFK cells. Therefore, they may be valuable candidates for the treatment of FHV-1 infection in cats.

## Background

Feline herpesvirus 1 (FHV-1), an *Alphaherpesvirus*, is one of the most common viruses among cats [1-3]. Infection is associated with respiratory disorders and ocular disease, including keratitis, conjunctivitis, corneal sequestration, keratoconjunctivitis and, ultimately, loss of sight [4,5]. The severity of clinical symptoms induced by FHV-1 infection can be reduced by vaccination [6,7]. However, especially in young kittens that have not yet reached the age of vaccination, but already lost maternal immunity, severe disease may still occur. Once these kittens have developed lesions, recurrent disease and virus excretion may occur upon reactivation at later age [8]. In order to control disease in these cases, treatment with an effective antiviral drug would be helpful.

Several antiviral drugs have already been tested for their efficacy to inhibit FHV-1 replication by means of a classical plaque number reduction assay. Based on the IC_50 _or concentration needed to reduce plaque number with 50 %, especially the acyclic nucleoside phosphonate (ANP) (S)-9-(3-hydroxy-2-phosphonylmethoxypropyl)adenine (HPMPA) [9] seems very efficient. Also, the ANP cidofovir [10] as well as the nucleoside analogues ganciclovir, penciclovir [10], idoxuridine [10,11], trifluridine [9,11] and vidarabine [11] are efficient in reducing the number of FHV-1-induced plaques *in vitro*. Acyclovir and foscarnet, although of proven value against human herpesviruses, have low efficacy against the formation of plaques by FHV-1 [9-11].

Besides by reducing the number of plaques, as described above, antiviral agents may also exert an effect by reducing the size of herpesvirus-induced plaques [12,13]. Reduction in plaque size may be a potential parameter for the ability of an antiviral agent to restrict the size of macroscopic lesions *in vivo *[12]. Up till now, no studies have addressed the efficacy of antiviral agents to limit the size of FHV-1-induced plaques.

The aim of the present study was to compare the efficacy of six antiviral drugs against FHV-1 *in vitro*. Parameters used to measure efficacy were the ability to limit FHV-1-induced plaque number (expressed as inhibitory concentration or IC _(NUMBER)_) as well as plaque size (expressed as IC _(SIZE)_) in Crandell-Rees feline kidney (CRFK) cells.

## Results

### Effect of the antiviral drugs on plaque number

**Figure 1:**
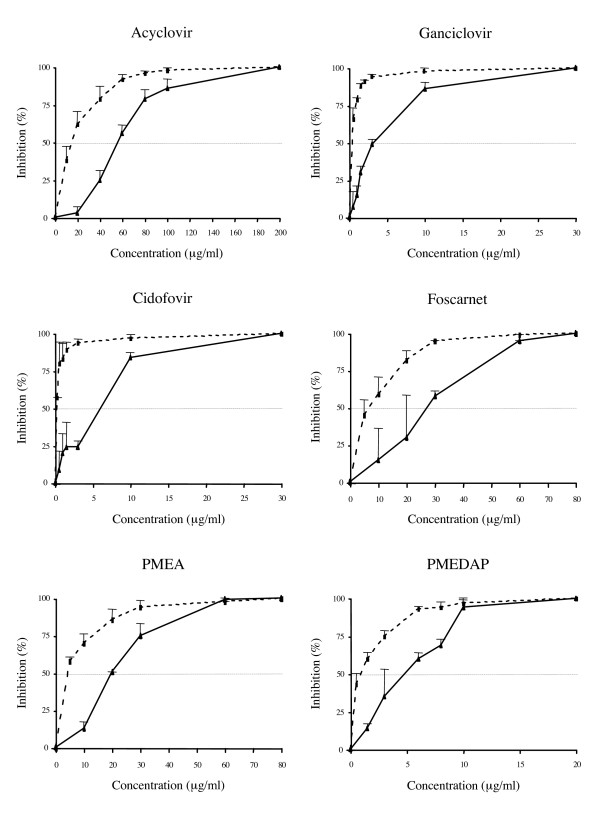
**Activity of six antiviral drugs against feline herpesvirus 1**. Dose-response curves are shown representing the activity of six antiviral drugs to reduce the number (solid line) and size (dashed line) of feline herpesvirus 1-induced plaques in Crandell-Rees feline kidney cells. Data are presented as the mean value of three independent experiments + standard deviation.

Figure 1 presents the dose-response curves regarding the inhibitory effect of each antiviral drug on plaque number (solid lines). The IC_50 (NUMBER) _values were extrapolated from the curves and are presented in Table 1. The capacity to reduce the number of plaques was most pronounced for ganciclovir, PMEDAP and cidofovir. Adefovir and foscarnet were intermediately efficient. Acyclovir was least efficient.

No significant variation in plaque number was observed between FHV-1-inoculated cells incubated with various concentrations of Roswell Park Memorial Institute (RPMI)-1640 medium and the untreated control (LSD, 95% confidence interval).

### Effect of the antiviral drugs on plaque size

It is known that marked differences may occur in plaque size when FHV-1 is grown *in vitro*. Therefore, data obtained in the plaque size assay were subjected to an F-test to examine whether the variation in plaque size between samples was significantly different from the variation in plaque size within a sample (95% confidence interval). Or, in other words, the F-test was used to answer the question whether variation in plaque size was merely an artefact or whether it was related to the use of antiviral drugs. It was found that all antiviral drugs exhibited a significant effect on plaque size (significance ≤ 0.001). Using a post-hoc LSD-test it was demonstrated that the reduction in plaque size was significant for all drugs at all concentrations when compared with the untreated control (95% confidence interval). The variation in plaque size observed in FHV-1-inoculated cells incubated with various concentrations of RPMI was not significantly different from the variation in plaque size observed in the untreated control (F-test, 95% confidence interval).

Figure 1 presents the dose-response curves regarding the inhibitory effect of each antiviral drug on plaque size (dashed lines). The IC_50 (SIZE) _values were extrapolated from the curves and are presented in Table 1. As observed for the reduction in plaque number, ganciclovir, PMEDAP and cidofovir were most potent in reducing plaque size. Adefovir and foscarnet were intermediately potent and acyclovir was least potent. For all six drugs, the IC_50 (SIZE) _was notably lower than the IC_50 (NUMBER) _(Figure 1; Table 1). The most remarkable effect was observed for cidofovir and ganciclovir.

### Effect of the antiviral drugs on the viability of CRFK cells

It was found that when CRFK cells were cultivated in the presence of 2% RPMI or more, that the relative viability of the cells was significantly lower in comparison with untreated CRFK cells incubated with complete medium (LSD, 95% confidence interval). In order to determine whether the antiviral products exerted a cytotoxic effect we, therefore, compared the mean relative viability of CRFK cells incubated with antiviral products with the mean relative viability of CRFK cells incubated with the corresponding concentration of RPMI (t-test, 95% confidence interval). Acyclovir exerted no significant effect on the viability of CRFK cells up till a concentration of 500 μg/ml (2220.2 μM), ganciclovir up till a concentration of 1000 μg/ml (3918.5 μM), cidofovir up till a concentration of 160 μg/ml (573.1 μM), foscarnet up till a concentration of 320 μg/ml (1666.7 μM), adefovir up till a concentration of 160 μg/ml (585.7 μM) and PMEDAP up till a concentration of 160 μg/ml (476.6 μM). These concentrations amply exceeded the IC_100 (NUMBER) _and the IC_100 (SIZE) _for all drugs (Figure 1), demonstrating that none of the products were toxic for CRFK cells at antiviral concentrations.

## Discussion

Over the years, an impressive array of antiviral agents has been developed for the treatment of human herpesvirus infections [14]. Many of these agents have already been studied for their efficacy against FHV-1 [15,16]. In the present study the efficacy of a selection of six antiviral drugs against FHV-1 was compared in CRFK cells using two different parameters, i.e. reduction in plaque number and reduction in plaque size.

Our study demonstrates that the efficacy of the drugs to reduce the number of plaques can be ranked as follows: ganciclovir (relative antiviral efficacy 1) → PMEDAP (relative antiviral efficacy 1.1) → cidofovir (relative antiviral efficacy 1.7) → adefovir (relative antiviral efficacy 5.9) → foscarnet (relative antiviral efficacy 11.2) → acyclovir (relative antiviral efficacy 19.9). The results for ganciclovir and cidofovir are similar to those obtained by Maggs and Clarke [10], who compared the ability of four antiviral drugs to reduce the number of plaques in CRFK cells. They found an IC_50 (NUMBER) _of 5.2 μM for ganciclovir and of 11 μM for cidovir (or a relative antiviral efficacy of 1 and 2.1, respectively). Concerning the activity of foscarnet and acyclovir, our results are slightly different from those obtained by Maggs and Clarke [10]. The latter authors found a lower activity for foscarnet (IC_50 (NUMBER) _232.9 μM or relative antiviral efficacy 44.8) and a higher activity for acyclovir (IC_50 (NUMBER) _57.9 μM or relative antiviral efficacy 11.1). Also, other authors obtained variable results on the activity of acyclovir against FHV-1, with IC_50 (NUMBER) _values ranging from 44.4 to 109 μM [9,11,17]. A potential explanation for the observed variation may be related to the virus strain. Indeed, in a comparative study of Nasisse et al. [11], the IC_50 (NUMBER) _for acyclovir in CRFK cells varied almost 2-fold depending on the FHV-1 strain used. Also, there may be inter-test variation involved. While both testing the activity of acyclovir against FHV-1 strain 727 on CRFK cells, Nasisse et al. [11] reported a markedly higher IC_50 (NUMBER) _(109 μM) than Maggs and Clarke [10] (57.9 μM).

Antiviral agents not only reduce the number of herpesvirus-induced plaques, they also contribute in the reduction of plaque size [12,13]. Our study is the first that addressed the ability of antiviral agents to reduce the size of FHV-1-induced plaques. Ranking the drugs following their relative ability to reduce plaque size resulted in cidofovir (1) → ganciclovir (2.4) → PMEDAP (3.9) → adefovir (20.9) → foscarnet (52) → acyclovir (95.1), which is an almost exactly equivalent ranking as that for plaque number data. The latter demonstrates that measuring plaque size is a useful and complementary means of assessing antiviral efficacy.

Interestingly, for all six drugs it was found that the concentration needed to reduce plaque size was notably lower than that needed to reduce plaque number (Figure 1; Table 1). The most remarkable effect was observed for cidofovir. Its IC_50 (SIZE) _was 30-times lower than its IC_50 (NUMBER)_. For ganciclovir, the IC_50 (SIZE) _was 8-times lower. For the other four drugs, a 4 to 5-times lower concentration was required to reduce plaque size with 50% when compared with the IC_50 (NUMBER)_. The relevance of this finding in view of protection of cats against FHV-1-induced disease remains to be determined. However, as already speculated by Mikloska and Cunningham [12], reduction of plaque size *in vitro *may be a potential parameter for the ability of an antiviral agent to restrict the size of virus-induced lesions *in vivo*.

The ability of adefovir to inhibit FHV-1 replication was found to differ markedly from PMEDAP. This seems rather surprising since both drugs, which belong to the class of acyclic nucleoside phosphonates, are closely related and share a similar antiviral mechanism that relies on the inhibition of viral DNA polymerase by the corresponding active diphosphate derivatives. A possible explanation could be a difference in uptake of the drugs by CRFK cells. However, Kramata and Downey [18] found that cellular uptake did not significantly differ between adefovir and PMEDAP in a human T-lymphoblastoid cell line CCRF-CEM. Alternatively, viral DNA polymerase may have a higher sensitivity for the PMEDAP diphosphate derivatives than for adefovir diphosphate derivatives, similar as described for cellular DNA polymerase [18]. Also, there may be a variation in intracellular stability of each drug metabolite as observed for other antiviral drugs [19,20]. It seems unlikely that the efficiency of phosphorylation by cellular enzymes and, consequently, the amounts of the active diphosphates differ. For both drugs, phosphorylation is catalyzed by mitochondrial and cytosolic isoenzymes of AMP kinase [21,22].

None of the products were toxic for CRFK cells at antiviral concentrations. However, the results of the cytotoxicity assay cannot simply be extrapolated to the cat itself, as sensitivity of host cells to a certain drug may differ from those of a continuous cell line. For example, while we were unable to detect a significant effect of cidofovir on the viability of CRFK cells up to a concentration of 160 μg/ml, Sandmeyer and colleagues [23] reported cytotoxic effects at a concentration of 50 μg/ml in a primary cell culture of feline corneal epithelial cells. Also, adefovir exerted no cytotoxic effects in our *in vitro *study, but when applied in cats for the treatment of feline infectious peritonitis virus [24] or feline leukaemia virus [25], severe haematological side effects were observed. This highlights the need for extensive safety studies in the cat.

## Conclusion

From our study, it can be concluded that measuring reduction in plaque number and plaque size are two valuable and complementary means of assessing the efficacy of an antiviral drug. By using these parameters for six selected antiviral drugs, we found that ganciclovir, PMEDAP, and cidofovir are most potent inhibitors of FHV-1 replication in CRFK cells. Therefore, they may be valuable candidates for the treatment of FHV-1 infection in cats.

## Methods

### Cells and virus

CRFK cells (CCL-94, ATCC, Manassas, VA, USA) were grown and maintained in complete medium consisting of Minimum Essential Medium (MEM) (Invitrogen, Merelbeke, Belgium) supplemented with 5 % fetal bovine serum, 100 U/ml penicillin, 0.1 mg/ml streptomycin, 0.1 mg/ml kanamycin, 0.3 mg/ml glutamin and 1 mg/ml lactalbumin hydrolysate. Cells were trypsinized once a week.

The Belgian strain 94K49 was used for inoculation. This strain was isolated from a cat with respiratory disorders and identified as FHV-1 using monoclonal antibodies 22C12, 22F4 and 41G4 against FHV-1 glycoprotein gC, gB, and gD, respectively [26]. Virus used for inoculation was at the 3^rd ^passage on CRFK cells.

### Antiviral drugs

Acyclovir, ganciclovir, adefovir and PMEDAP were dissolved in RPMI-1640 medium (Invitrogen, Merelbeke, Belgium) at a concentration of 1 mg/ml. Cidofovir and foscarnet were dissolved in RPMI-1640 at a concentration of 2 mg/ml. Subsequently, all drugs were filtered through a 0.45 μm filter to obtain a sterile solution and stored at 4°C. Before use, acyclovir was adapted to room temperature to re-dissolve possible precipitates.

### Antiviral assays

CRFK-cells were grown to confluency in 24-well culture plates (Nunc A/S, Roskilde, Denmark) in complete medium at 37°C and 5 % CO_2_. Then, medium was removed and cells in each well were inoculated with 40 PFU of FHV-1 in 200 μl of complete medium. After 1 h incubation at 37°C and 5 % CO_2_, cells were washed twice with MEM. Afterwards, they were overlaid with medium containing carboxymethylcellulose (CMC) (0.94 %) and various concentrations of antiviral drugs. First, a broad range of concentrations was tested to allow a preliminary estimation of the antiviral efficacy. Then, final concentrations were chosen, as indicated in Figure 1. FHV-1-inoculated cells incubated with CMC and complete medium without antiviral drugs were included as an untreated control. Additionally, FHV-1-inoculated cells incubated with CMC and various concentrations of RPMI, ranging from 0.25 % to 20 % in complete medium, were included to test whether RPMI influenced plaque formation by FHV-1.

At 72 hours post inoculation, overlay medium was removed, cells were rinsed with PBS and, consecutively, fixed with paraformaldehyde 4 % and methanol + 1 % H_2_O_2_. An immunoperoxidase monolayer assay (IPMA) was performed based on van der Meulen *et al*. [27]. Briefly, cells were incubated with a mixture of monoclonal antibodies 22C12, 22F4 and 41G4 against FHV-1 glycoprotein gC, gB, and gD, respectively [26] for 1 h at 37°C. After three washing steps, peroxidase-labelled goat anti-mouse IgG (Molecular Probes) were added for 1 h at 37°C. Following three additional washing steps, substrate solution of 3-amino-9-ethylcarbazole in 0.05 M acetate buffer with 0.05 % hydrogen peroxide was added. Cells were incubated for 10 minutes at 37°C and then, substrate solution was replaced with acetate buffer to block enzymatic staining.

The number of FHV-1-induced plaques was counted for each concentration of antiviral drug, for each concentration of RPMI and for untreated control samples using light microscopy (Olympus IX50). The inhibitory effect of the antiviral drugs on plaque number was calculated by following formula:

Percentage inhibition(NUMBER)=(1−(plaque number)antiviral(plaque number)control)×100
 MathType@MTEF@5@5@+=feaafiart1ev1aaatCvAUfKttLearuWrP9MDH5MBPbIqV92AaeXatLxBI9gBaebbnrfifHhDYfgasaacH8akY=wiFfYdH8Gipec8Eeeu0xXdbba9frFj0=OqFfea0dXdd9vqai=hGuQ8kuc9pgc9s8qqaq=dirpe0xb9q8qiLsFr0=vr0=vr0dc8meaabaqaciaacaGaaeqabaqabeGadaaakeaacqqGqbaucqqGLbqzcqqGYbGCcqqGJbWycqqGLbqzcqqGUbGBcqqG0baDcqqGHbqycqqGNbWzcqqGLbqzcqqGGaaicqqGPbqAcqqGUbGBcqqGObaAcqqGPbqAcqqGIbGycqqGPbqAcqqG0baDcqqGPbqAcqqGVbWBcqqGUbGBdaWgaaWcbaGaeiikaGIaeeOta4KaeeyvauLaeeyta0KaeeOqaiKaeeyrauKaeeOuaiLaeiykaKcabeaakiabg2da9iabcIcaOiabigdaXiabgkHiTmaalaaabaGaeiikaGIaemiCaaNaemiBaWMaemyyaeMaemyCaeNaemyDauNaemyzauMaeeiiaaIaemOBa4MaemyDauNaemyBa0MaemOyaiMaemyzauMaemOCaiNaeiykaKIaemyyaeMaemOBa4MaemiDaqNaemyAaKMaemODayNaemyAaKMaemOCaiNaemyyaeMaemiBaWgabaGaeiikaGIaemiCaaNaemiBaWMaemyyaeMaemyCaeNaemyDauNaemyzauMaeeiiaaIaemOBa4MaemyDauNaemyBa0MaemOyaiMaemyzauMaemOCaiNaeiykaKIaem4yamMaem4Ba8MaemOBa4MaemiDaqNaemOCaiNaem4Ba8MaemiBaWgaaiabcMcaPiabgEna0kabigdaXiabicdaWiabicdaWaaa@96B5@

The minimal concentration of each product required to reduce the plaque number with 50 % (IC_50 (NUMBER)_) was hand-calculated from the dose-response curves generated from the data.

The size of FHV-1-induced plaques for each concentration of antiviral drug, for each concentration of RPMI and for untreated control samples was determined as follows. Plaques were photographed using light microscopy (Olympus IX50) and a digital camera (Sony Progressive 3CCD), attached to a Macintosh computer. Plaques were randomly selected in each sample and the number counted varied between a minimum of 3 and a maximum of 17 (mean number counted per sample was 10.4 ± 2.5). Then, the size of each of the plaques was determined in pixels using Scion 1.63 (National Institutes of Health) and, finally, the mean size per sample was calculated. The inhibitory effect of the antiviral drugs on plaque size was subsequently calculated by following formula:

Percentage inhibition(SIZE)=(1−(plaque size)antiviral(plaque size)control)×100
 MathType@MTEF@5@5@+=feaafiart1ev1aaatCvAUfKttLearuWrP9MDH5MBPbIqV92AaeXatLxBI9gBaebbnrfifHhDYfgasaacH8akY=wiFfYdH8Gipec8Eeeu0xXdbba9frFj0=OqFfea0dXdd9vqai=hGuQ8kuc9pgc9s8qqaq=dirpe0xb9q8qiLsFr0=vr0=vr0dc8meaabaqaciaacaGaaeqabaqabeGadaaakeaacqqGqbaucqqGLbqzcqqGYbGCcqqGJbWycqqGLbqzcqqGUbGBcqqG0baDcqqGHbqycqqGNbWzcqqGLbqzcqqGGaaicqqGPbqAcqqGUbGBcqqGObaAcqqGPbqAcqqGIbGycqqGPbqAcqqG0baDcqqGPbqAcqqGVbWBcqqGUbGBdaWgaaWcbaGaeiikaGIaee4uamLaeeysaKKaeeOwaOLaeeyrauKaeiykaKcabeaakiabg2da9iabcIcaOiabigdaXiabgkHiTmaalaaabaGaeiikaGIaemiCaaNaemiBaWMaemyyaeMaemyCaeNaemyDauNaemyzauMaeeiiaaIaem4CamNaemyAaKMaemOEaONaemyzauMaeiykaKIaemyyaeMaemOBa4MaemiDaqNaemyAaKMaemODayNaemyAaKMaemOCaiNaemyyaeMaemiBaWgabaGaeiikaGIaemiCaaNaemiBaWMaemyyaeMaemyCaeNaemyDauNaemyzauMaeeiiaaIaem4CamNaemyAaKMaemOEaONaemyzauMaeiykaKIaem4yamMaem4Ba8MaemOBa4MaemiDaqNaemOCaiNaem4Ba8MaemiBaWgaaiabcMcaPiabgEna0kabigdaXiabicdaWiabicdaWaaa@8F2F@

The minimal concentration of each product required to reduce the plaque size with 50 % (IC_50 (SIZE)_) was hand-calculated from the dose-response curves generated from the data.

For each experiment, three independent replicates were performed.

### Cytotoxicity assay

In order to determine whether the antiviral drugs exerted a toxic effect on CFRK cells, a neutral red assay was performed. This assay is based on the incorporation of the supravital dye neutral red into living cells. In brief, CRFK-cells were grown to confluency in 96-well culture plates (Nunc A/S, Roskilde, Denmark) in complete medium at 37°C and 5 % CO_2_. Then, medium was removed, cells were incubated with various concentrations of antiviral drugs ranging from 5 to 500 μg/ml in medium and cultured for 72 h at 37°C and 5 % CO_2_. Cells incubated for 72 h with complete medium lacking antiviral drugs were included as viable controls. Cells successively incubated with complete medium for 70 h and distilled water for 2 h were included as non-viable controls. Additionally, cells incubated for 72 h with various concentrations of RPMI, ranging from 0.25% to 50% in complete medium, were included to test whether RPMI influenced the viability of CRFK cells.

After incubation, 50 μl of neutral red solution (0.1% in distilled water) was added to each well and plates were incubated at 37°C and 5% CO_2 _for 1 h. Then, cells were rinsed with phosphate-buffered salt solution and air-dried at room temperature for 1 h. Finally, 50 μl of SDS (10% in distilled water) and 100 μl HC1 (0.2 M) were added and plates were shaken for 15 sec. Absorbance was measured on a Multiskan RC (Thermo Labsystems) using a 492-nm filter. Viability of cells was calculated by following formula:

Percentage of viable cells=ODt−ODdODc−ODd×100
 MathType@MTEF@5@5@+=feaafiart1ev1aaatCvAUfKttLearuWrP9MDH5MBPbIqV92AaeXatLxBI9gBaebbnrfifHhDYfgasaacH8akY=wiFfYdH8Gipec8Eeeu0xXdbba9frFj0=OqFfea0dXdd9vqai=hGuQ8kuc9pgc9s8qqaq=dirpe0xb9q8qiLsFr0=vr0=vr0dc8meaabaqaciaacaGaaeqabaqabeGadaaakeaacqqGqbaucqqGLbqzcqqGYbGCcqqGJbWycqqGLbqzcqqGUbGBcqqG0baDcqqGHbqycqqGNbWzcqqGLbqzcqqGGaaicqqGVbWBcqqGMbGzcqqGGaaicqqG2bGDcqqGPbqAcqqGHbqycqqGIbGycqqGSbaBcqqGLbqzcqqGGaaicqqGJbWycqqGLbqzcqqGSbaBcqqGSbaBcqqGZbWCcqGH9aqpdaWcaaqaaiabd+eapjabdseaejabdsha0jabgkHiTiabd+eapjabdseaejabdsgaKbqaaiabd+eapjabdseaejabdogaJjabgkHiTiabd+eapjabdseaejabdsgaKbaacqGHxdaTcqaIXaqmcqaIWaamcqaIWaamaaa@63E9@

where *ODt *is the absorbency of cells incubated with antiviral drugs or RPMI, *ODd *the absorbency of the non-viable cell control and *ODc *the absorbency of the viable cell control, respectively.

For each concentration of antiviral drug, for each concentration of RPMI and for control samples, eight wells were tested per experiment. Three independent replicates of each experiment were performed.

### Statistical analysis

Statistical analysis was based on analysis of variance (ANOVA) using SPSS (SPSS Inc., Chicago, Illinois, USA).

## Abbreviations

ANOVA: analysis of variance

ANP: acyclic nucleoside phosphonate

CMC: carboxymethylcellulose

CRFK: Crandell-Rees feline kidney

FHV-1: feline herpesvirus 1

HPMPA: (S)-9-(3-hydroxy-2-phosphonylmethoxypropyl)adenine

IPMA: immunoperoxidase monolayer assay

MEM: Minimum Essential Medium

PMEDAP: 9-(2-phosphonylmethoxyethyl)-2,6-diaminopurine

RPMI: Roswell Park Memorial Institute

## Authors' contributions

KVDM participated in the design of the study as well as the coordination and performance of the experiments and drafted the manuscript. BG was involved in the performance of the experiments. SC and HN participated in the design of the study and reviewed the manuscript.

## Figures and Tables

**Table 1:** The effect of selected antiviral drugs on number and size of feline herpesvirus 1-induced plaques in Crandell-Rees feline kidney cells

**Antiviral drug**	**IC_50 (NUMBER)_**	**IC_50 (SIZE)_**
Acyclovir	56 μg/ml (248.7 μM)	15 μg/ml (66.6 μM)
Ganciclovir	3.2 μg/ml (12.5 μM)	0.4 μg/ml (1.7 μM)
Cidofovir	6 μg/ml (21.5 μM)	0.2 μg/ml (0.7 μM)
Foscarnet	27 μg/ml (140.6 μM)	7 μg/ml (36.4 μM)
Adefovir	20 μg/ml (73.2 μM)	4 μg/ml (14.6 μM)
PMEDAP	4.8 μg/ml (14.3 μM)	0.9 μg/ml (2.7 μM)

IC_50 (NUMBER)_: concentration of a product required to reduce the plaque number with 50 % in comparison with untreated cells

IC_50 (SIZE)_: concentration of a product required to reduce the plaque size with 50% in comparison with untreated cells

PMEDAP: 9-[2-phosphonylmethoxyethyl]-2, 6-diaminopurine
